# First estimates of Greenland shark (*Somniosus microcephalus*) local abundances in Arctic waters

**DOI:** 10.1038/s41598-017-19115-x

**Published:** 2018-01-17

**Authors:** Brynn M. Devine, Laura J. Wheeland, Jonathan A. D. Fisher

**Affiliations:** Centre for Fisheries Ecosystems Research, Fisheries and Marine Institute of Memorial University of Newfoundland, 155 Ridge Road, St. John’s, NL A1C 5R3 Canada

## Abstract

Baited remote underwater video cameras were deployed in the Eastern Canadian Arctic, for the purpose of estimating local densities of the long-lived Greenland shark within five deep-water, data-poor regions of interest for fisheries development and marine conservation in Nunavut, Canada. A total of 31 camera deployments occurred between July-September in 2015 and 2016 during joint exploratory fishing and scientific cruises. Greenland sharks appeared at 80% of deployments. A total of 142 individuals were identified and no individuals were observed in more than one deployment. Estimates of Greenland shark abundance and biomass were calculated from averaged times of first arrival, video-derived swimming speed and length data, and local current speed estimates. Density estimates varied 1–15 fold among regions; being highest in warmer (>0 °C), deeper areas and lowest in shallow, sub-zero temperature regions. These baited camera results illustrate the ubiquity of this elusive species and suggest that Nunavut’s Lancaster Sound eco-zone may be of particular importance for Greenland shark, a potentially vulnerable Arctic species.

## Introduction

One of very few polar shark species, the Greenland shark *Somniosus microcephalus* is found throughout the cold waters of the North Atlantic and Arctic Oceans^1^. It is the largest fish in the Arctic and a top predator^2–4^, despite anomalously slow swimming speeds^5^ and presumed limited visual acuity as a common host to the corneal copepod parasite *Ommatokoia elongata*^6^. However, the Greenland shark remains a poorly studied species and many aspects of its basic ecology are unknown^3^. Limited life history studies have revealed a remarkably slow growth rate (<1 cm yr^−1^ ^7^), late maturation timing (mature females >450 cm^2^ and ~134 years old^8^), and Greenland shark currently holds the record for the longest lifespan of any vertebrate species (>272 years)^8^.

Body size^9^ and survival to maturity^10^ are key traits of elasmobranchs associated with population extinction risks worldwide. The paucity of data concerning these traits and Greenland shark population dynamics has led to its designation as ‘near threatened’^11^ or ‘data deficient’^12^ throughout parts of its range; in other areas it remains unassessed^13^. Therefore an urgent need exists to address major knowledge gaps concerning past, present, and potential future population dynamics^13^. While some other shark species’ abundance and biomass baselines are being monitored and revised^14,15^, similar fishery-independent baselines for Greenland shark have not yet been established in any area.

Much of our current understanding regarding the distribution and abundance of Greenland sharks has been obtained from historical commercial exploitation and current bycatch in northern fisheries. Historically, this species was commercially fished for liver oil until 1960^16^, with annual catch estimates in the early 20^th^ century ranging from 32,000 to 150,000 sharks in Greenland and Norway^17,18^. The species is still harvested today for human and sled-dog consumption, with mean annual reported landings of 47 t since 1980^19^. It is also a bycatch species in northern Canadian fisheries, particularly within Greenland halibut *Reinhardtius hippoglossoides* trawl and gillnet fisheries, with mean annual bycatch rates from 1996 to 2015 in Canada’s NAFO divisions 0AB exceeding 105 t per year^13,20^. However, in areas of the North Atlantic and Arctic Ocean where directed shark fishing has not occurred – such as the waters of the Canadian Arctic Archipelago – the geographic and bathymetric range of this species remains largely unknown.

Scientific longline surveys are the most common fishery-independent survey method used for sampling shark populations. Relative abundance estimates (e.g. catch per unit effort) provide insights into the spatial and temporal variability in shark abundance and habitat use^21,22^. However, such surveys are not ideal for all species because mortality rates can be high and even capture stress can have adverse effects which may result in reduced fitness and/or delayed post-capture mortality^23,24^. Although quantitative estimates of capture mortality rates for Greenland shark have yet to be enumerated, this species is prone to gear entanglement as these large sharks rotate to free themselves from bottom longline gear, and other Greenland sharks are known to depredate conspecifics caught in this way^25^. These behaviours could exacerbate stress-related impacts and may increase the likelihood of capture mortality^13,26^, therefore alternative methods to scientific longlining are needed to quantify Greenland shark abundance and distribution.

Optical technologies are utilized worldwide to survey marine organisms, providing versatile, non-destructive tools to monitor both benthic and pelagic species^27–29^. In particular, baited remote underwater video (BRUV) surveys have become increasingly popular as cost-effective and relatively simple survey methods, with high accessibility to users as many BRUVs can be readily assembled with inexpensive components^30,31^. BRUVs have produced results comparable to some traditional fishing gear based survey methods, including longline surveys sampling relative shark abundances^32,33^. BRUVs have also proven useful in surveying sensitive habitats such as marine protected areas^34,35^, reef habitats^36,37^, and other habitats where the low impact nature of BRUVs are deemed favourable^38,39^. With comparatively fewer commercial fisheries occurring in the Arctic Ocean^40,41^, many benthic marine ecosystems have been spared the impacts of bottom trawling, presumably preserving pristine benthic habitats including cold-water corals and sponges. BRUVs therefore provide an ideal method to survey polar marine environments while maintaining the integrity of these sensitive habitats.

BRUVs generate many types of data that can be used to characterize benthic habitats, assess functional diversity, body sizes, and animal behaviours, and quantify the relative abundances and distributions of identified species. Priede and Merrett^42^ demonstrated a significant positive relationship between fish abundances from trawl surveys and arrival time of the first fish to proximately deployed baited cameras for the abyssal grenadier *Coryphaenoides armatus*. This discovery confirmed the validity of a model used to estimate local theoretical abundance from baited cameras using first arrival time, current velocity, and swimming speed^42,43^, and has since been applied to other species^44–46^. Greenland shark individuals are believed to be non-shoaling, mobile predators, and are known opportunistic scavengers^47,48^. Therefore, while the theoretical abundance model was originally developed for an abyssal teleost and not a Selachimorphan, search strategies are presumably comparable between species, although how species-specific differences in olfactory sensitivities may influence theoretical density estimates requires further research^29^. As Greenland shark satisfies these behavioural assumptions for the model, BRUVs may provide a non-destructive and efficient method of generating local population estimates for this poorly understood Arctic predator.

**Figure 1:**
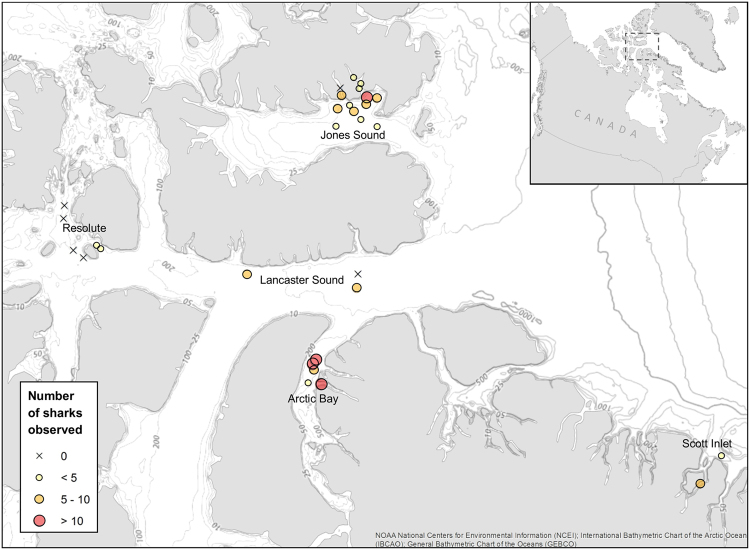
Map of baited camera deployments where Greenland sharks were observed, with symbol sizes proportional to the number of individuals distinguished from each set. The ‘X’ indicates sets where no sharks were observed. Map created using ArcGIS® software (ArcMap™ v.10.3.1 by ESRI®) and reference maps from the NOAA National Centers for Environmental Information. For more information about Esri® software, visit www.esri.com.

Here we present estimates of Greenland shark local relative abundances within the Canadian Arctic Archipelago using data obtained from baited camera surveys and established models of theoretical abundance using first arrival times and bottom current and swimming speed estimates. We present these estimates in the context of local habitat and oceanographic conditions, relative abundance, and size-and-sex structure of Greenland sharks observed in the northern Nunavut regions of Arctic Bay, Resolute, Lancaster Sound, Scott Inlet and Grise Fiord (Fig. 1).

## Results

**Figure 2:**
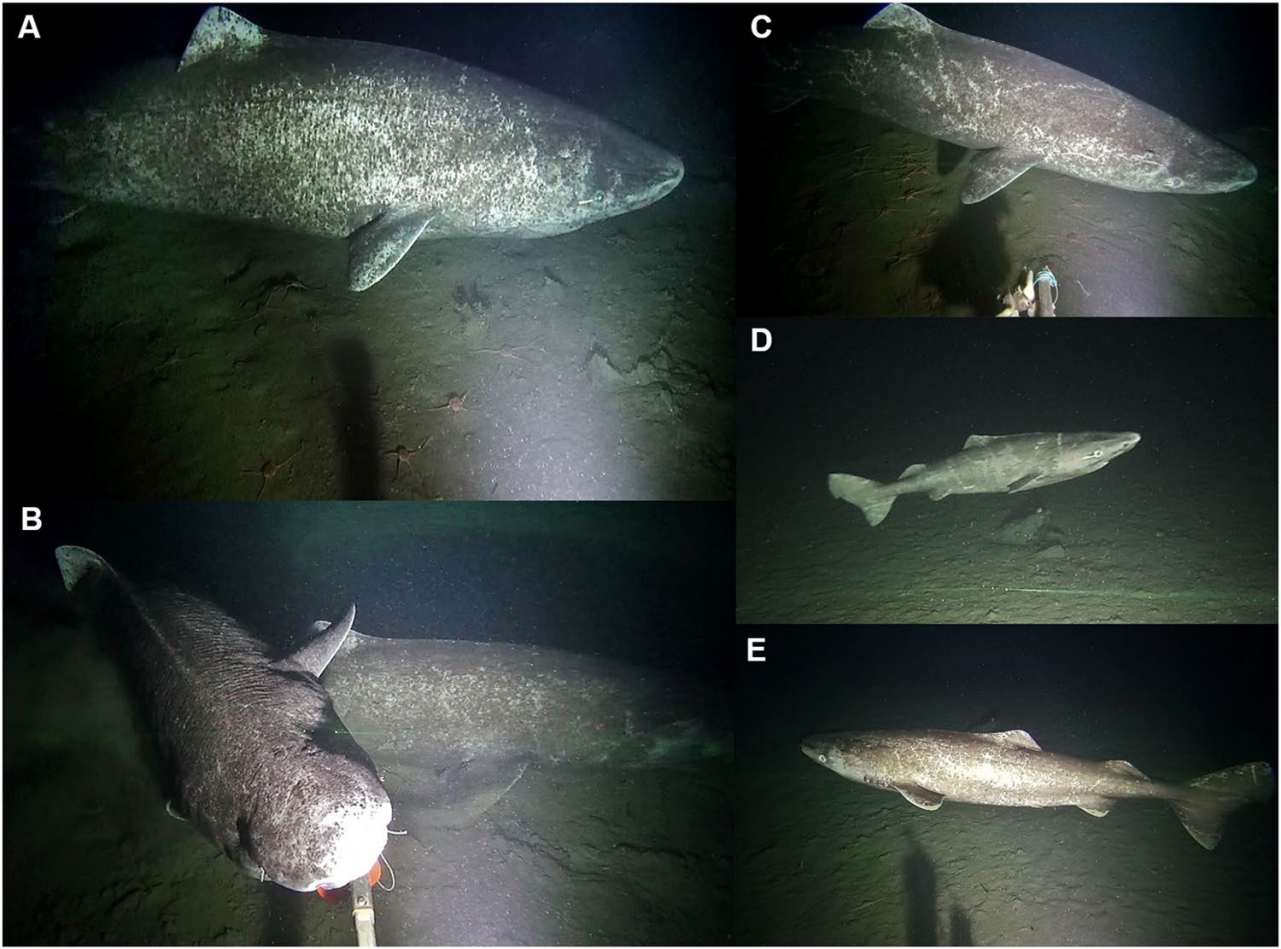
Images of Greenland sharks attracted to the baited camera: (**A**) Typical size and coloration of sharks observed, showing distinct scar patterns; (**B**) Feeding on squid bait, with multiple sharks within field of view; (**C**) Example of unique scar patterns used to distinguish individuals; (**D** and **E**) Juvenile sharks <150 cm observed in Scott Inlet.

**Figure 3:**
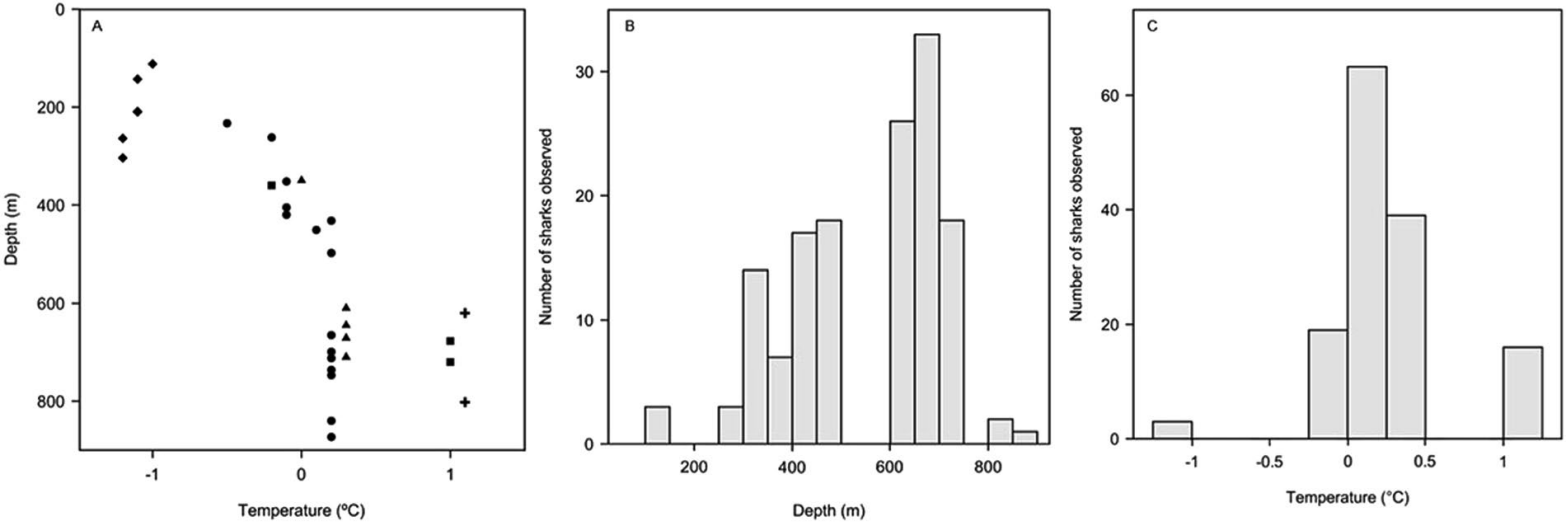
Depth versus temperature relationships for deployments (**A**) from all regions: Arctic Bay (▲), Lancaster Sound (▪), Resolute (◆), Jones Sound (●), and Scott Inlet (+), with frequency distributions for the number of sharks observed across sampled depths (**B**) and temperatures (**C**).

**Figure 4:**
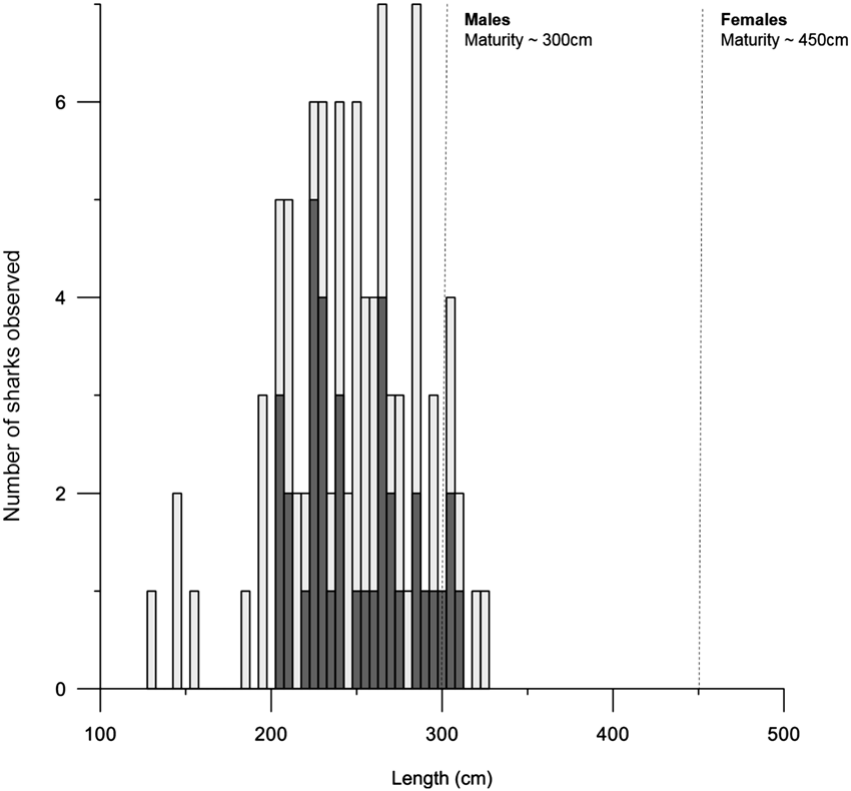
Length-frequency distribution of Greenland sharks measured from camera footage (n = 93), with males (dark grey bars) and females (light grey bars). Dashed lines indicate proposed maturity lengths for both sexes^2^.

Greenland shark was the primary consumer of the bait at the camera and was present in 25 of 31 deployments, but with differing local densities among regions (Table 1). In total, 142 individuals were identified from the video footage (Fig. 2) and no individuals were observed in multiple camera sets. Sharks were present - and often numerous - in all sets near Arctic Bay, with up to 18 individuals present in a single set in Admiralty Inlet (Fig. 1, Table S1). Observation rates based on the number of individuals sighted per hour were highest in Arctic Bay (mean = 1.1 sharks hr^−1^), similar among Lancaster Sound, Scott Inlet, and Jones Sound (mean = 0.5, 0.5, and 0.6 sharks hr^−1^, respectively), and were lowest in sets near Resolute (mean = 0.1 sharks hr^−1^) with only a few observations (n = 3) of sharks in the two shallowest sets within Resolute Pass (Fig. 1). Approximately 75% of sharks were observed at temperatures from 0 to 0.5 °C (Fig. 3A) and at depths from 450 to 800 m (Fig. 3B). A general linear model indicated no significant length differences (n = 93 sharks measured) between males versus females (Fig. 4). Overall, no differences in size or sex ratios of sharks were observed among locations (F_9,83_ = 1.93, p = 0.06) with the exception of Scott Inlet, which had a significantly higher proportion of small (<150 cm) sharks (p < 0.01) in the set that occurred within Scott Inlet fiord. Despite body lengths as high as 325 cm, most male and all female sharks were below hypothesised sizes at maturity (Fig. 4).

**Table 1:** Summary of mean length and associated weights derived from the MacNeil *et al*.^3^ length-weight relationship, theoretical abundances per square kilometer, theoretical biomass per square kilometer, mean number of sharks observed in the first 250 minutes of each deployment, and mean total number of sharks observed in each deployment per region.

Region	Mean length (cm) ± S.D.	Estimated weight (kg)	Abundance estimate (#-km^−2^)	Biomass estimate (kg-km^−2^)	Mean observed in first 250 minutes	Mean observed total
Arctic Bay	254.5 ± 32.0 (n = 25)	170.4	5.0	852.0	4	11
Jones Sound	249.5 ± 33.2 (n = 52)	160.2	4.7	752.9	1	4
Lancaster Sound	235.6 ± 47.8 (n = 7)	133.8	1.6	214.1	2	2
Resolute	281.3 ± 28.3 (n = 3)	233.2	0.4	93.3	0	1
Scott Inlet	198.3 ± 73.8 (n = 6)	78.1	15.5	1210.6	3	6

First arrival times of Greenland sharks to the bait differed among regions, with mean arrival times (±S.D.) longest in Resolute (280 min ± 84) compared to 198 min (±142) in Jones Sound, 191 min (±142) in Scott Inlet, and 118 min in both Arctic Bay (±146) and Lancaster Sound (±112) (Table S1). Even prior to estimating local theoretical abundances, there was a negative exponential relationship between first arrival times and total individuals observed (N = 9.50e^−0.004t^, R^2^ = 0.52, Fig. S1). A generalized linear model also found a significant relationship between total number of sharks observed and first arrival time (z = −3.396, p < 0.001) and set duration (z = 2.331, p = 0.02), but not with region or temperature (p > 0.06). Bait was removed by Greenland sharks from Set-1 within 22 minutes of arrival on the seafloor, which may have lessened attraction throughout the deployment, therefore this set was excluded from local abundance calculations. Swimming speeds derived from 31 measurements across 20 Greenland sharks (TL range 185–314 cm) in this study resulted in a mean swimming speed of 0.27 ms^−1^ (S.D. = 0.07; range 0.15–0.42 ms^−1^) and no significant correlation between shark length and speed (r = 0.11, p = 0.65; Fig. S2). Bottom current speed estimates extracted from a regional ocean model varied between locations, with considerably higher velocities in Lancaster Sound and Resolute (0.1 ms^−1^) compared to other regions (0.02–0.05 ms^−1^).

**Figure 5:**
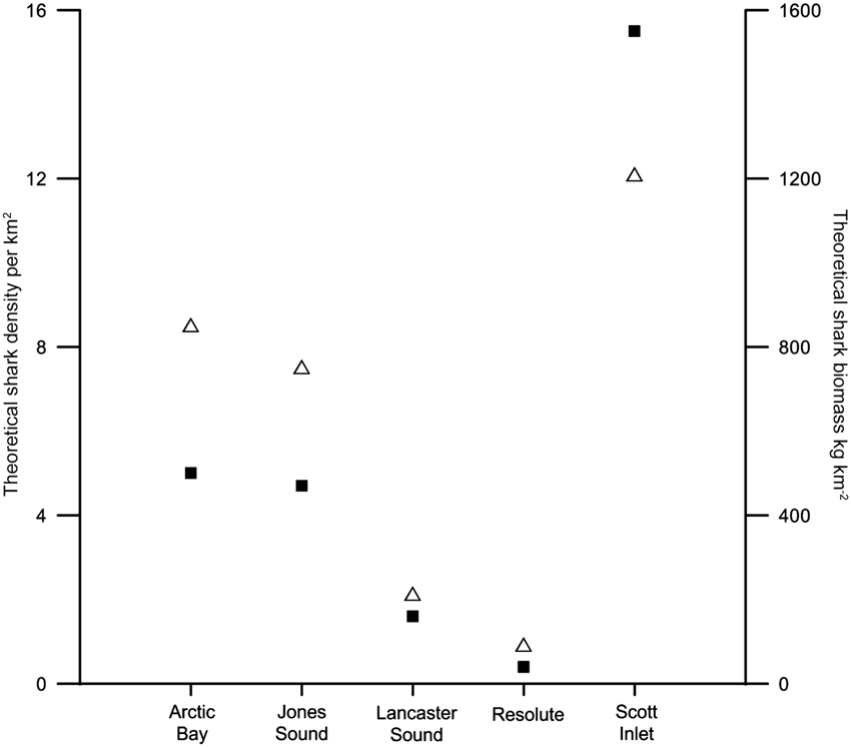
Comparison of theoretical abundance (▪) and biomass (Δ) estimates for all five sampled regions. Abundance estimates were calculated using the model validated by Priede and Merrett^43^ using mean first arrival time and bottom current speed estimates within region, and a mean swimming speed of 0.27 ms^−1^ for all regions. Biomass estimates were calculated from an established Greenland shark length-weight relationship^3^ using video-derived mean lengths per region.

Local abundance estimates using mean first arrival times within region, mean swimming speed, and mean bottom current speed within region indicated variable theoretical abundance values between regions. Shark density estimates were higher in Arctic Bay (5.0 individuals km^−2^) and Jones Sound (4.7 individuals km^−2^) regions compared to waters of Lancaster Sound (1.6 individuals km^−2^) and Resolute (0.4 individual km^−2^) (Fig. 5). Local estimates for Scott Inlet were highest (15.5 individuals km^−2^), but we note that only 2 sets occurred in this region. Estimated local biomass values showed the same rankings as abundances among regions, ranging from 93 to 1210 kg km^−2^ estimated across regions based on numbers and sizes observed (Table 1).

## Discussion

This study provides the first data and estimates of Greenland shark local and regional abundances independent of fishing and bycatch estimates. This finding is a first step toward fulfilling a major knowledge gap currently preventing assessment of population status needed for the management of this species^13^. Theoretical abundance estimates derived from first arrival times of Greenland sharks and total individuals observed both indicate higher concentrations in Arctic Bay, Jones Sound, and Scott Inlet, suggesting these regions may be of particular importance for this species during the summer months. Additionally, camera deployments provided a simple means to collect data on depth, temperature, shark size, and sex distribution in poorly sampled areas within the range of Greenland sharks in Canadian waters.

While Greenland sharks were observed in 80% of camera deployments, spatial variation in their observed and estimated local densities and biomass were associated with co-varying oceanographic conditions. In regions of the Canadian Arctic Archipelago examined, there is a strong summer thermocline, such that water temperatures typically reach minima at intermediate depths (ca. 100–150 m) and become warmer at depth^49^. Such changes are evident within and among regions, with shark densities peaking at intermediate temperatures sampled (Fig. 3A), and at depths between 450–800 m (Fig. 3B). This may explain the lower number of sharks observed and estimated for waters near Resolute, where average set temperature was −1.1 °C and depths below 450 m are unavailable. Although there is variation in sharks observed among sets within regions, with future sampling and a more stratified or systematic spatial coverage, it may be possible to extrapolate to larger areas. In our sampled areas, Admiralty Inlet has an area of 8557 km^2^, Lancaster Sound spans 26335 km^2^, McDougal Sound covers 4327 km^2,^^50^; Jones Sound is approximately 14,330 km^2^. In all of these regions, large areas cover the depth and temperature ranges sampled in this study. Given the opportunistic deployments confined here to the areas of interest to exploratory fishing, the wide-spread occurrences of Greenland sharks across regions highlights their apparent ubiquity, with local abundances influenced by temperature and depth.

**Figure 6:**
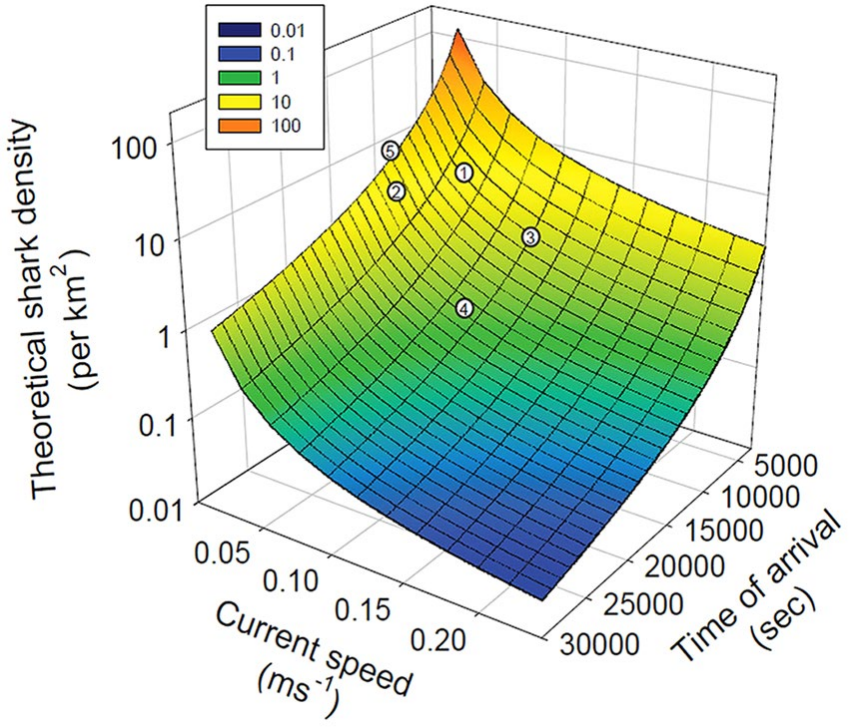
Surface plot of variation in theoretical shark density as functions of varying mean current speed and mean time of first arrival, using data from five regions and mean swimming speed of 0.27 ms^−1^. Individual numbered points correspond to Arctic Bay (‘1’), Jones Sound ‘2’, Lancaster Sound ‘3’, Resolute ‘4’, and Scott Inlet ‘5’. Note that given current speed and fish swimming speed have the same effect and weighting on the model (see Methods), the effect of current or swim speed can be generalized from this illustration.

Both the sensitivity of our estimated shark densities to model inputs and the need for validation of modeled density estimates are required before such values could be used to estimate population abundances at any spatial scale. In order to demonstrate the effects of first arrival time and current speed, we explored variation in theoretical shark densities across a range of both parameters, and overlaid results from the five regions examined (Fig. 6). The inverse square relationship dictates average t_0_ values should be used in abundance models for each region^42^, however the influence of current speed (Fig. 6) emphasizes the need to consider spatial and temporal variation in current speeds^51^. The effects of variable swimming speed are equal to those of current speeds in this model, and while we used a fixed swimming speed based on the mean of our observations (0.27 ms^−1^), our value is within the range of mean reported swimming speed for this species (0.22 ms^−1^ to 0.34 ms^−1^ based on ultrasonic tracking^52^ and data logging tags^5^, respectively). Replacing our video-derived speed with the only direct measure of speed for Greenland shark (mean = 0.34 ms^−1^) derived from accelerometer tagging data^5^, the calculated theoretical densities change slightly, decreasing between 0.1–0.3 sharks per km^2^ among regions.

Further validation of our model results might be achieved through comparisons with data sets within this study and those using other techniques. Within our video analyses, there was a strong positive correlation between the total number of individuals observed and local densities based on first arrival times among 4 of our 5 sampling regions (r = 0.97, p = 0.02, n = 4), with the exception of Scott Inlet where number of individuals observed did not align with theoretical estimates. As one of the two sets in Scott Inlet was characterized by a low mean current speed and quick first arrival time resulting in an unusually high abundance, additional sets are required for more robust density estimates in this region. However, the general correspondence between abundance metrics provides confidence in the theoretical estimates, as the mean number of sharks actually sighted within regions (but not used directly in estimates of local density) corresponds with the proposed abundance values for most regions (Table 1), but also highlights the need for further studies to determine the necessary sampling effort within regions.

Tagging studies have begun to elucidate movement patterns within the eastern Canadian Arctic^52,53^ and other Arctic regions^53,54^, indicating Greenland sharks are capable of long distance migrations (>1000 km) with excursions between inshore and offshore waters. As these sharks may be highly migratory, seasonal fluctuations in local densities may occur. More camera deployments are needed to examine intra- and inter-annual variability in shark abundance and habitat use. However, even our 31 deployments demonstrate clear differences in relative abundances between regions, and highlight water readily used by Greenland sharks in summer months, including potential nursery areas for small (<150 cm) sharks. Given the recent establishment of the boundaries of what will become the Lancaster Sound National Marine Conservation Area which encompasses nearly half of our camera deployments, further surveys are essential to characterize spatial variation in local densities and connectivity between broader Arctic regions, and to provide new information for species management both inside and outside of protected areas.

Our findings are revealing in the context of recent life history information and future management potential for this species within the Canadian Arctic. Assuming similar growth rates as individuals sampled from other regions and examined for maturity status^2^, the males and females we observed may all be sexually immature. While these may be somewhat small (mean 2.48 m, SD = 0.40) relative to mature sharks (Fig. 4), the mean length among 166 sharks of known and unknown sex compiled previously^55^ was 3.07 m (SD = 0.73). Together, these findings suggest that the vast majority of reported specimens of this species may be juveniles. In addition the use of superficial markings to distinguish individuals provided us with a catalog of over 100 individuals appearing in videos throughout the sampled area. As with other shark species where photo-ID catalogues exist (e.g. white shark *Carcharadon carcharias*, whale shark *Rhincodon typus*), with repeat deployments this information could potentially be used to track individuals throughout their range^56–58^. With adequate coverage and seasonality to deployments, BRUV surveys could additionally help to describe movement patterns and site fidelity behaviour which for this species remain largely unknown.

In a comparative context, it is also revealing to examine our Greenland shark video survey results from unexploited regions to estimates of dominant shark local density and biomass from intensively sampled, pristine tropical areas. Recently revised estimates of grey reef shark *Carcharhinus amblyrhynchos* abundance from the protected island of Palmyra have revealed mean shark densities of 21.3 sharks km^−2^ ^14^. That estimate is comparable to the upper end of estimated Greenland shark abundances (Fig. 5), illustrating similar densities between these two dominant sharks in their respective tropical and Arctic ecosystems. Estimated biomass of the grey reef shark based on their mean lengths^15^ (718 kg km^−2^ at Palmyra^59^) are lower than Greenland shark estimates within three regions (Table 1), due to large differences in mean mass per individual. However, total biomass of sharks at Palmyra^14^ greatly exceeds that of any and all Arctic locations, given the presence of multiple shark species at Palmyra. A further comparison of our results to BRUV survey data from the remote tropical island of New Caledonia show surprising similarities in observation rates. There, shark occurrences from 209 BRUV deployments yielded an observation rate of 0.43 individuals hr^−1^ for all 9 reef sharks combined^60^, compared to our mean Greenland shark observation rate of 0.56 individuals hr^−1^ from our five Arctic regions. The size and apparent density of Greenland sharks in Canadian Arctic waters conceals the fact that as the only large fish predator they have a unique taxonomic and functional role in Arctic waters compared to shark species in many other areas.

Finally, our results illustrate that in areas explored within the Canadian Arctic Archipelago, Greenland sharks are seemingly widespread and commonly inhabit a wide range of depth and temperature conditions. However, as with other shark species^9,10^, their life history features concomitantly highlight the need for considering Greenland sharks in spatial management and bycatch avoidance plans in this region. In gillnet fisheries targeting Greenland halibut, Greenland shark bycatch was negatively associated with halibut catch, suggesting that where possible, shark avoidance and maximum targeted catch rates may be mutually achievable goals^61^. Whether similar patterns occur in longline fisheries has yet to be established. Spatial management has multiple approaches and recently, the Lancaster Sound region has been identified by Parks Canada, Nunavut communities, and non-governmental organizations as a priority conservation region and is expected to be designated Canada’s largest area of protected ocean. Our study provides a largely non-invasive means to evaluate marine conservation areas before and after establishment using baited underwater video.

## Material and Methods

### Baited Camera

A total of 31 baited camera deployments were conducted in July-September of 2015 and 2016 aboard the 30 m Arctic Fishery Alliance vessel *Kiviuq I* in the following five regions within the northern Canadian territory of Nunavut: Admiralty Inlet and Adams Sound near the community of Arctic Bay (hereafter ‘Arctic Bay’); central Lancaster Sound; southeast McDougall Sound/Barrow Strait near the community of Resolute (hereafter ‘Resolute’); eastern Jones Sound including Starnes Fiord and Grise Fiord (hereafter ‘Jones Sound’), and; Scott Inlet (Fig. 1). Deployment locations were selected to provide maximum spatial, depth, and habitat coverage throughout each region within the confined range of the exploratory fisheries (largely for Greenland halibut) simultaneously conducted aboard the vessel. Deployment depths varied between sites, ranging from 112 to 850 m (Table S1) reflecting differences in bathymetry among these regions (Fig. 3). Bottom temperatures at each camera set were derived from temperature loggers (DST centi-TD Star-Oddi, Gardabaer, Iceland) attached to the nearest bottom fishing gear set conducted at similar depth within Resolute and Arctic Bay regions (2015) or attached directly to the camera frame (2016). At camera deployments in 2015 where temperature loggers were unavailable (i.e. three Lancaster Sound deployments), bottom temperature was taken from CTD (conductivity, temperature, depth) profiler casts performed aboard the CCGS Amundsen in August 2015^62^ at similar depths and at locations nearest (<50 nm) to the camera deployments.

The baited camera lander consisted of a single high-definition camera with integrated reference lasers (parallel and spaced 6.2 cm apart) and a white light source (1Cam Alpha, Aquorea LED; SubC Imaging Inc., Clarenville, Newfoundland and Labrador, Canada) mounted to a weighted aluminum frame tethered to a surface buoy for later retrieval. The camera was positioned at the top of the frame at 1.6 m above the seafloor and oriented downward and outward at approximately a 60° angle, with continuous recording at each location. A horizontal bait arm was positioned 50 cm above the seafloor, extended toward the field of view, with approximately 2 kg of commercial grade squid bait (6–8 whole squid) affixed to the bait arm for each deployment.

Within each camera set, arrival times were recorded for the first Greenland shark individual to appear after the camera frame landed on the seafloor, and for each subsequent individual arriving to the baited camera. Individual Greenland sharks were easily distinguished using unique markings (i.e. scar patterns and coloration), length, and sex, which enabled quantification of numbers of individuals observed per set (Fig. 2). Shark lengths were estimated from video still images using the software ImageJ^63^ for all sharks that fully entered the field of view and were in line with the camera reference lasers as required for accurate estimates of body size. A general linear model was used to test for differences in length between sexes (Table S2) and location. An additional generalized linear model with a poisson distribution was used to examine the relationship between the total number of sharks observed and parameters of region, first arrival time, duration, and temperature across deployments. All analyses were performed using the statistical software R version 3.3.2^64^. All methods were carried out under experimental licenses and ethics approval granted by the Department of Fisheries and Oceans Canada and in accordance with experimental protocol approved by the animal ethics committee of Memorial University of Newfoundland.

### Abundance estimates

Densities of Greenland shark within the 5 regions were calculated using first arrival time (t_0_, seconds), shark swimming speed (Vf, ms^−1^), and current velocity (Vw, ms^−1^) based on the following equation originally developed and validated for the abyssal grenadier^43^:

1 $${\rm{N}}(\#\,{\rm{individuals}}\,{{\rm{km}}}^{-2})=0.3849{(1/{{\rm{V}}}_{{\rm{f}}}+1/{{\rm{V}}}_{{\rm{w}}})}^{2}/{{{\rm{t}}}_{0}}^{2}$$

Estimates of abundance were calculated from averaged first arrival times within region^42^ and using mean measures of swimming speed and current speed. Mean swimming speed for this species was derived from the subset of shark encounters (n = 31) where swimming occurred at a consistent rate perpendicular to the camera view, and with lasers passing horizontally along the anteroposterior axis of each shark. No measurements were taken from sharks while approaching the bait, only sharks passing through the field of view at a steady swimming speed. Still images from videos were processed in ImageJ software to measure the speed lasers moved alongside the body, providing inputs to calculate mean swimming speed for the present study. Estimates of bottom current velocity were extracted from the Ocean Navigator portal (http://navigator.oceansdata.ca) using the Regional Ice Ocean Predication System 2016 data set^65,66^. For each set, the location, date, and time of deployment were used as model filters to obtain an average bottom current speed for each set. As we could not measure shark mass, biomass estimates within each region were derived using a length-weight relationship^3^ and our mean shark length per region to calculate estimates of kg km^−2^.

### Ethics

Deployment of baited cameras and fishing gear during this study were conducted under scientific licenses S-15/16-1041-NU, S-16/17-1017-NU and animal use protocol approvals FWI-2015-052, FWI-2016-029 granted by the Department of Fisheries and Oceans Canada, and animal ethics approval by Memorial University of Newfoundland research animal care File no. 20170278.

## Electronic supplementary material

Supplementary Figures
