# Supplementary material for: First estimates of Greenland shark (Somniosus microcephalus) local abundances in Arctic waters
Source: Sci Rep. 2018 Jan 17;8:974. doi: 10.1038/s41598-017-19115-x (PMC5772532; doi:10.1038/s41598-017-19115-x)
Supplement: Supplementary file 1 — Supplementary Figures [file 41598_2017_19115_MOESM1_ESM.pdf]

SUPPLEMENTARY MATERIALS

First estimates of Greenland shark (*Somniosus microcephalus*) local abundances in Arctic waters

Brynn M. Devine*, Laura J. Wheeland, Jonathan A.D. Fisher

Centre for Fisheries Ecosystems Research, Fisheries and Marine Institute of Memorial University of Newfoundland, 155 Ridge Road, St. John's, NL A1C 5R3, Canada

*Corresponding author email: brynn.devine@mi.mun.ca

Figure S1. Comparison of number of individuals observed vs. first arrival time of sharks in each camera deployments for all regions: Arctic Bay (▲), Lancaster Sound (▪), Resolute (♦), Jones Sound (•), and Scott Inlet (+).

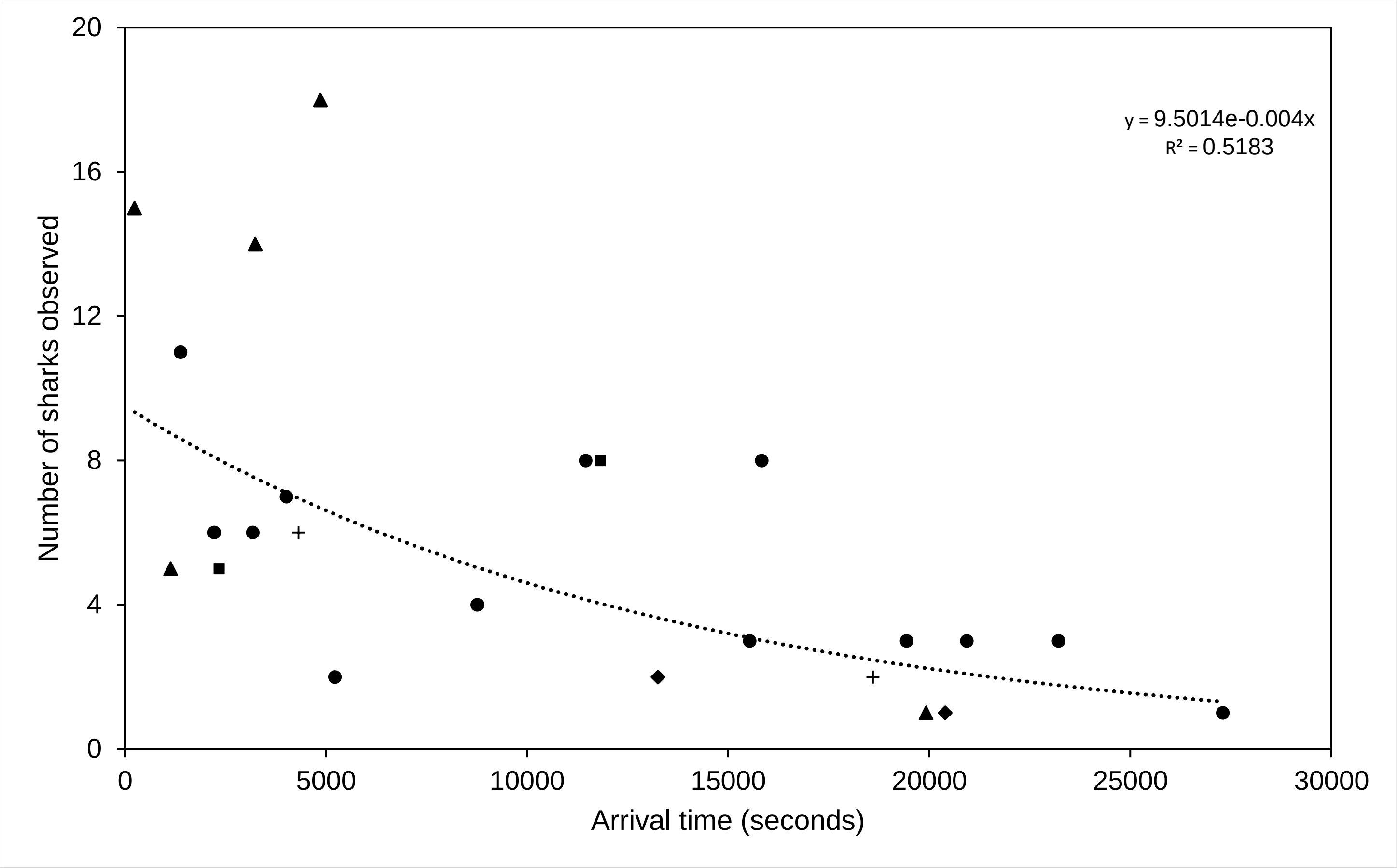

Table S1. Summary of camera deployment details and shark arrival times, number of sharks present in first 250 minutes of each deployment, and total number of sharks throughout each duration. Number of individuals was not reported for Set 25 ('-') where camera did not remain upright for the entirety of the set so quantification of individuals was not possible; 'X's indicate sets were no sharks were observed.

Set ID	Region	Date	Latitude N	Longitude W	Depth (m)	Temperature (°C)	Duration (min)	First arrival t_0 (min)	Sharks in first 250 minutes	Total number of sharks
**1**	Arctic Bay	21-Aug-15	73.176	-85.679	610	0.3	570	19	4	5
**2**	Arctic Bay	22-Aug-15	73.245	-85.714	645	0.3	595	4	4	15
**3**	Arctic Bay	23-Aug-15	73.292	-85.563	671	0.3	587	81	7	18
**4**	Lancaster Sound	24-Aug-15	74.108	-83.497	677	1.0	615	197	2	8
**5**	Arctic Bay	25-Aug-15	72.996	-85.417	350	0.0	605	54	4	14
**6**	Arctic Bay	26-Aug-15	73.026	-85.961	710	0.3	386	332	0	1
**7**	Lancaster Sound	27-Aug-15	74.276	-83.369	720	1.0	495	X	X	X
**8**	Lancaster Sound	31-Aug-15	74.369	-88.369	360	-0.2	462	39	5	5
**9**	Resolute	02-Sep-15	75.101	-96.999	304	-1.2	250	X	X	X
**10**	Resolute	04-Sep-15	74.625	-95.075	112	-1.0	448	221	1	2
**11**	Resolute	05-Sep-15	74.497	-95.848	209	-1.1	450	X	X	X
**12**	Resolute	06-Sep-15	74.941	-96.970	264	-1.2	599	X	X	X
**13**	Resolute	08-Sep-15	74.574	-96.352	210	-1.1	414	X	X	X
**14**	Resolute	09-Sep-15	74.662	-95.292	143	-1.1	395	340	0	1
**15**	Jones Sound	26-Jul-16	76.312	-82.784	665	0.2	464	387	0	3
**16**	Jones Sound	27-Jul-16	76.238	-82.623	736	0.2	568	191	3	8
**17**	Jones Sound	28-Jul-16	76.504	-82.143	420	-0.1	487	324	0	3
**18**	Jones Sound	29-Jul-16	76.649	-82.416	262	-0.2	570	259	0	3
**19**	Jones Sound	30-Jul-16	76.565	-82.076	352	-0.1	505	87	1	2
**20**	Jones Sound	31-Jul-16	76.381	-81.857	451	0.1	565	23	6	11
**21**	Jones Sound	01-Aug-16	76.357	-81.345	498	0.2	565	67	2	7
**22**	Jones Sound	02-Aug-16	76.303	-81.955	747	0.2	532	37	3	6
**23**	Jones Sound	03-Aug-16	76.123	-82.333	873	0.2	553	455	0	1
**24**	Jones Sound	04-Aug-16	76.015	-81.546	712	0.2	571	349	0	3
**25**	Jones Sound	06-Aug-16	76.086	-82.775	840	0.2	176	134	-	-
**26**	Jones Sound	07-Aug-16	76.080	-83.582	699	0.2	581	146	1	4
**27**	Jones Sound	08-Aug-16	76.297	-83.432	432	0.2	568	264	0	8
**28**	Jones Sound	09-Aug-16	76.448	-83.153	405	-0.1	406	53	3	6
**29**	Jones Sound	10-Aug-16	76.542	-83.168	233	-0.5	447	X	X	X
**30**	Scott Inlet	22-Sep-16	70.890	-71.599	620	1.1	549	72	5	6
**31**	Scott Inlet	23-Sep-16	71.123	-70.530	802	1.1	570	310	0	2

Figure S2. Comparison of shark length (TL cm) versus swimming speed (ms^{-1}) derived from video measurements (n=31) from 20 individuals. For individuals where multiple measurements were taken, swimming speeds were averaged for each individual. Data of length versus swimming speed from two additional studies[5, 52] have been added for comparison.

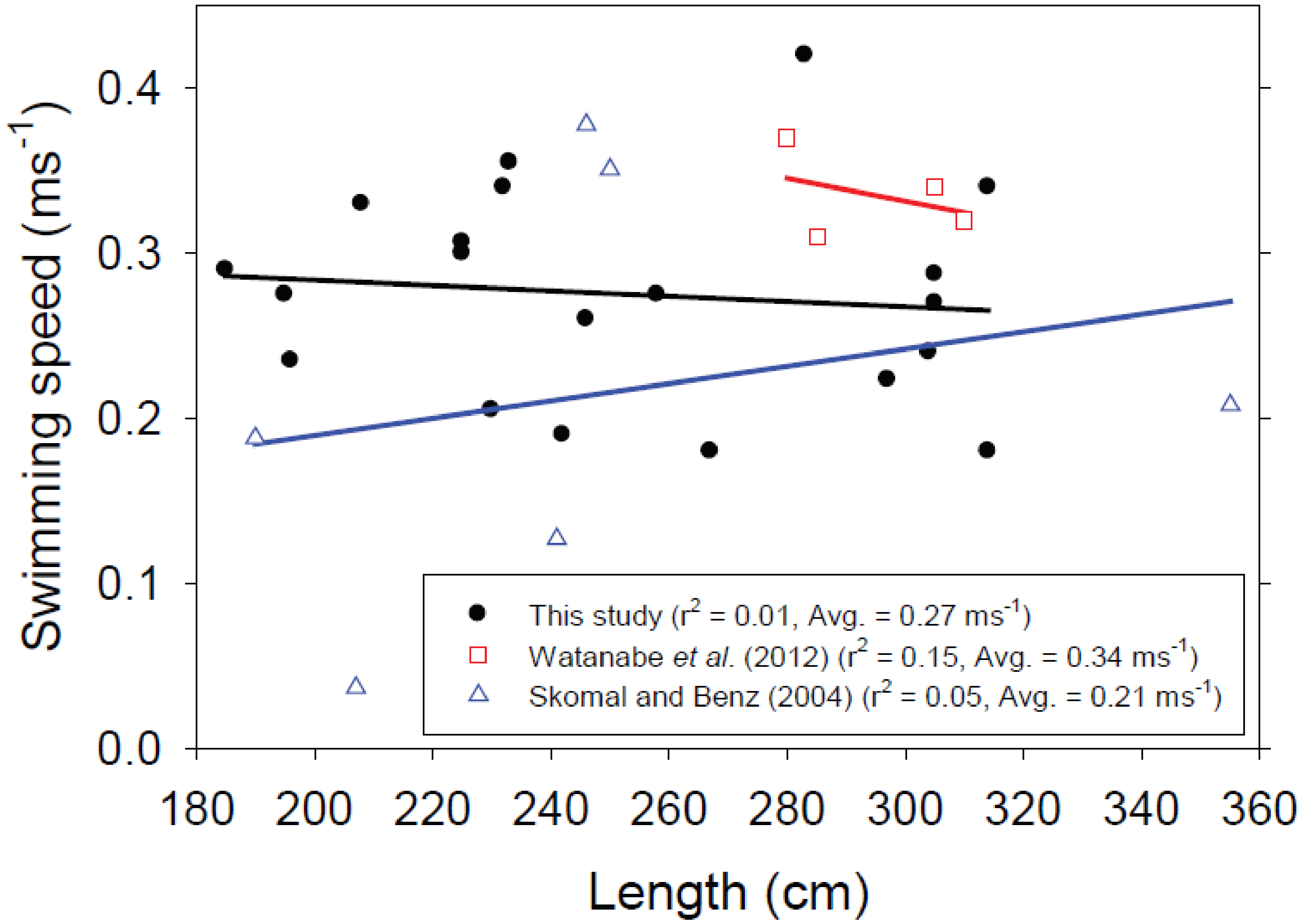

Table S2. Length (TL in cm) and sex of individuals observed from videos and used in comparison of sex and size between sampling regions. Not included are individuals where only sex but no length were recorded.

Region	Total Length (cm)	Sex
Jones Sound	210	F
Jones Sound	250	F
Jones Sound	206	M
Jones Sound	250	F
Jones Sound	265	M
Jones Sound	248	F
Jones Sound	240	M
Jones Sound	285	F
Jones Sound	275	M
Jones Sound	305	F
Jones Sound	285	M
Jones Sound	205	M
Jones Sound	215	F
Jones Sound	263	F
Jones Sound	270	M
Jones Sound	195	F
Jones Sound	210	M
Jones Sound	230	M
Jones Sound	250	M
Jones Sound	255	F
Jones Sound	238	F
Jones Sound	270	F
Jones Sound	210	M
Jones Sound	205	M
Jones Sound	225	M
Jones Sound	288	M
Jones Sound	290	M
Jones Sound	235	M
Jones Sound	320	F
Jones Sound	256	M
Jones Sound	212	F
Jones Sound	210	F
Jones Sound	306	M
Jones Sound	283	F
Jones Sound	288	F
Jones Sound	233	M
Jones Sound	276	F

Jones Sound	233	F
Jones Sound	259	F
Jones Sound	250	F
Jones Sound	225	M
Jones Sound	196	F
Jones Sound	221	M
Jones Sound	295	F
Jones Sound	220	F
Jones Sound	224	M
Jones Sound	228	F
Jones Sound	240	F
Jones Sound	275	F
Jones Sound	305	F
Jones Sound	252	F
Jones Sound	295	M
Scott Inlet	225	M
Scott Inlet	146	F
Scott Inlet	131	F
Scott Inlet	218	F
Scott Inlet	145	F
Scott Inlet	325	F
Arctic Bay	253	F
Arctic Bay	297	F
Arctic Bay	225	M
Arctic Bay	240	F
Arctic Bay	230	M
Arctic Bay	242	M
Arctic Bay	240	F
Arctic Bay	270	M
Arctic Bay	260	F
Arctic Bay	208	F
Arctic Bay	305	M
Arctic Bay	285	F
Arctic Bay	285	F
Arctic Bay	268	M
Arctic Bay	229	M
Arctic Bay	246	F
Arctic Bay	232	F
Arctic Bay	258	F
Arctic Bay	304	M
Arctic Bay	314	M

Arctic Bay	205	F
Arctic Bay	240	M
Arctic Bay	267	F
Arctic Bay	195	F
Arctic Bay	265	M
Lancaster Sound	157	F
Lancaster Sound	260	F
Lancaster Sound	185	F
Lancaster Sound	266	F
Lancaster Sound	230	M
Lancaster Sound	286	F
Lancaster Sound	265	F
Resolute	266	M
Resolute	314	F
Resolute	264	M
